# Removal of organic matter and nutrients from hospital wastewater by electro bioreactor coupled with tubesettler

**DOI:** 10.1038/s41598-022-12166-9

**Published:** 2022-06-03

**Authors:** Roohul Abad Khan, Rachida El Morabet, Nadeem A Khan, Sirajuddin Ahmed, Majed Alsubih, Nabisab Mujawar Mubarak, Mohammad Hadi Dehghani, Rama Rao Karri, Nooshin Zomorodiyan

**Affiliations:** 1 Department of Civil Engineering, King Khalid University, Abha, Saudi Arabia; 2 Department of Geography, FLSH, LADES-M, Hassan II University of Casablanca, Mohammedia, Morocco; 3 Department of Civil Engineering, Jamia Millia Islamia, New Delhi, India; 4 Petroleum and Chemical Engineering, Faculty of Engineering, Universiti Teknologi Brunei, Bandar Seri Begawan, BE1410 Brunei Darussalam; 5 Department of Environmental Health Engineering, School of Public Health, Tehran University of Medical Sciences, Tehran, Iran; 6 Institute for Environmental Research, Center for Solid Waste Research, Tehran University of Medical Sciences, Tehran, Iran; 7 Department of Organic Chemistry, College and Faculty of Chemistry, University of Science and Technology, Tehran, Iran

**Keywords:** Ecology, Environmental sciences

## Abstract

Wastewater consisting of different pharmaceuticals and drug residues is quite challenging to treat and dispose of. This situation poses a significant impact on the health aspect of humans and other biotic organisms in the environment. The main concern of hospital wastewater (HWW) is the resistivity towards treatment using the different conventional methods. For the treatment of HWW, this study was performed using an electro bioreactor using hospital wastewater. The electro reduction overcomes the effect of toxic elements in hospital wastewater, and biodegradation removes organic matter and nutrients from wastewater. This study investigated electro bioreactor performance for treating hospital wastewater connected with tubesettler. The parameters of chemical oxygen demand, nitrate, and phosphate concentration were analyzed to evaluate an influent and effluent from electro bioreactor and tubesettler. Also, Kinetic modelling for chemical oxygen demand, nitrate, and phosphate removal was done. The chemical oxygen demand was reduced by 76% in electro bioreactor, and 31% in tubesettler, 84%. The nitrate and phosphate were reduced within permissible discharge limits with a final effluent concentration of 1.4 mg L^−1^ and 3 mg L^−1^. Further studies are required to assess the impact of pharmaceutical compounds in hospital wastewater on the system's performance.

## Introduction

Hospital wastewater (HWW) is of growing concern as it constitutes elements toxic to the environment. Treatment methods for HWW have been gaining attention in recent research due to their pharmaceutical contents^1–5^. Stringent wastewater standards render conventional wastewater treatment systems inefficient^6^. Also, the need to treat specific wastewater from various industries and other origins further augments the problem. This leads to a desire for innovative and new technologies to meet the required standards^7,8^. Among different wastewater technologies, particular focus is given to electro bioreactors (EBR). The degradation of pollutants primarily depends on the availability of electrons in the system^9^. The electro biological system overcomes this shortage of electron donors. It acts as electrochemical assistance for the microbiological system to reduce pollutants from wastewater. The cathode can continuously provide electron (hydrogen production) and electric fields with low reduction potential. EBRs utilize electric energy for treating wastewater. The primary four mechanisms in EBR are electrocoagulation (EC), electrodepositions (ED), electrooxidation (EO), and electro flotation (EF). Recovery from wastewater stream is achieved through an electromagnetic deposition. EC is used for wastewater treatment, and EF effectively separates flocculated sludge from wastewater. EO is primarily employed for reducing organic matter, refractory pollutants, and nutrients from wastewater^10^.

EBR has various applications for treating wastewater, from raw municipal wastewater^11^ to landfill leachate^12–15^. It has also been examined to treat specific chemical compounds in wastewater, such as reducing 2,4-dicholorophenoxyacetic acid, degradation of tetracycline, degradation of antibiotics, and reducing refractory organic pollutants^14,16^. Also, EBR has been employed for reducing membrane fouling for treating wastewater^6,17^. EBR has been used to treat wastewater combined with submerged membrane^18–20^ and as an electroperoxin treatment process^14,21,22^. Despite wide application in wastewater treatment studies^23–25^, EBR performance evaluation for HWW treatment is still lacking. This is primarily due to its limited applications combined with other techniques that support treatment systems rather than fully perform individual treatment systems. Hence, this study was carried out to investigate the performance of EBR as a particular treatment system for HWW.

A tubesettler combined with EBR was used in this experiment. This overcame the shortcoming of the combination study and gave an insight into the treatment efficiency of EBR as an individual treatment system. The objectives of this study are to:

i. Investigate chemical oxygen demand (COD) reduction in hospital wastewater using EBR and tubesettler.
ii. Determine the removal efficiency of nutrients, i.e., nitrate and phosphate.
iii. Compare the removal efficiency of EBR and tubesettler to assess its suitability and validate it as an effluent treatment unit.

## Materials and methods

### Hospital wastewater sampling

The hospital wastewater used in this study was obtained from the Guru Teg Bahadur Hospital wastewater treatment plant during March 2021 to January 2022, with a 600 m^3^/day capacity in New Delhi, India. All collected samples were transported to the Environmental laboratory at Mewat Engineering College, Nuh, Haryana, India-122107, and stored at 4 °C before being used as influent in EBR and connected tubesettler. Before conducting experiments, these samples were taken out of the refrigerator to reach room temperature (20–25 °C) before use.

### Experimental setup

**Figure 1:**
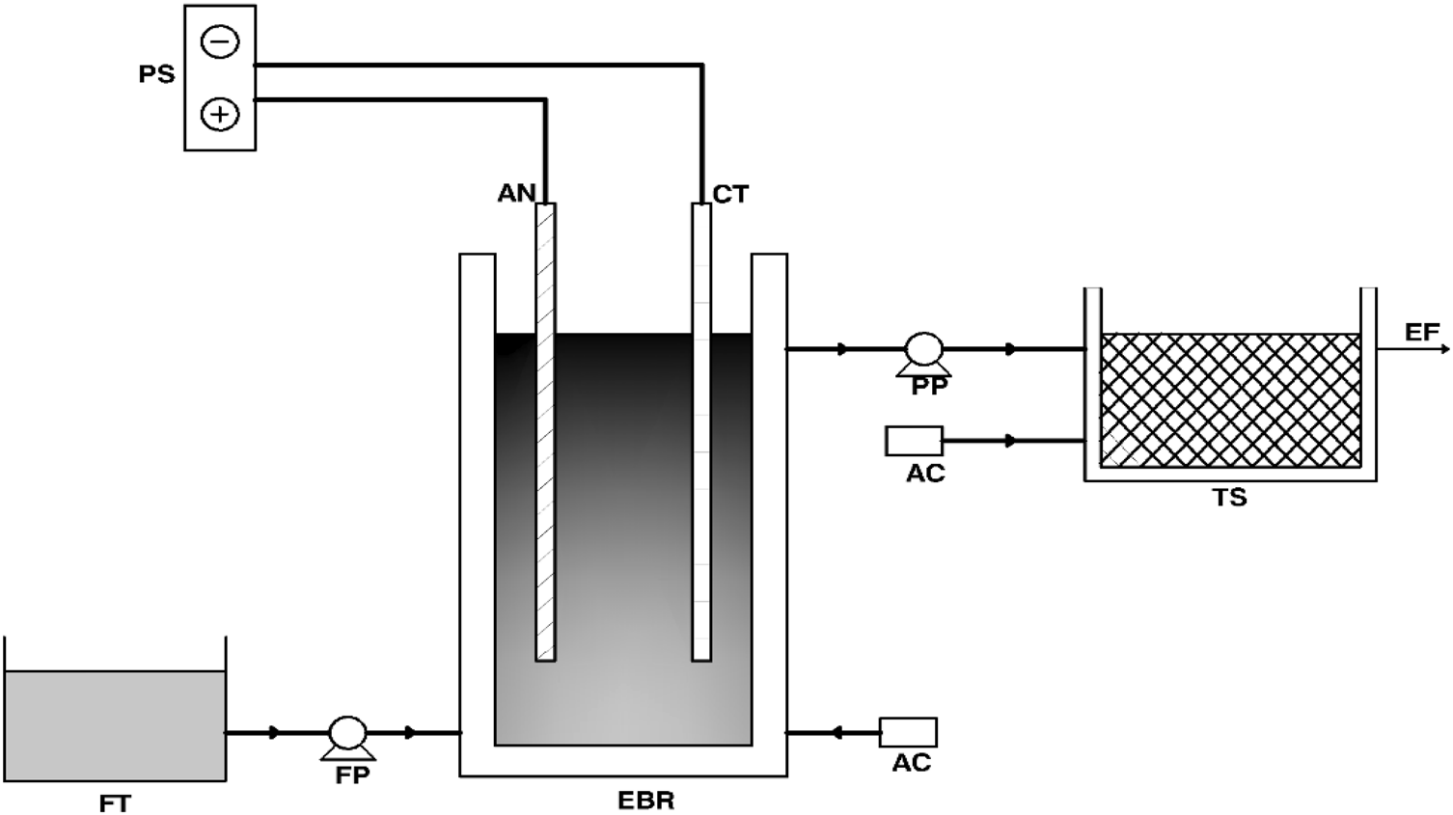
Schematic diagram of setup used in experiment (FT = Feed tank, FP = Feed Pump, EBR = Electro bioreactor, PS = Power Supply, AN = Anode, CT = Cathode, PP = Peristatic Pump, AC = Air Compressor, TS = tubesettler, EF = Effluent).

A laboratory-scale experimental setup was designed and installed for this study, as shown in Fig. 1. The Setup comprised of electro bioreactor connected in series with a tubesettler. The working volume of the electro bioreactor was 14.2 L. The effluent from EBR and tubesettler was obtained via a peristaltic pump. The constant volume in the reactor was maintained using a level sensor connected to the feeding pump. The anode and cathode had an area of 100 cm^2^ with a spacing of 5.7 cm. DC power supply was maintained at a 1 V/cm gradient. Continuous aeration was provided in both EBR and tubesettler. The influent wastewater characteristics and operating conditions used are presented in Tables 1 and 2.

**Table 1:** Influent parameters detected in EBR along with tubesettler.

Parameters	Unit	Range
COD	mg L^−1^	200–400
Biological Oxygen Demand (BOD)	mg L^−1^	560–1250
Turbidity	NTU	460–550
Total Suspended Solids (TSS)	mg L^−1^	2450–3900
Alkalinity	mg L^−1^ of CaCO_3_	760–1230
pH	–	6.1–7.8

**Table 2:** Operating conditions for EBR.

S.no	Characteristics	Period (days)			
		0–20	20–30	30–40	50–60
1	Cycle (h)	24	20	18	12
2	Loading rate (kg COD/m^3^/day)	0.28	1.25	2.25	3.45
3	COD removal (%)	82	65	74	80
4	Current timing (10 milliamperes)	20	30	40	50
5	SVI (ml/g MLSS)	114–124	104	106	70–80
6	MLSS (g/L)	2.1–3.1	3.2–4.2	4.2–6.2	6.2–7.2

### Laboratory analysis

EBR and tubesettler's performance was evaluated based on the pollutant's concentration in the effluent. Influent and effluent samples were taken from EBR and tubesettler and analyzed for COD, nitrate, and phosphate concentration removal as standard methods^26,27^. HACH DR 2800 was used for diagnosing the concentration of parameters. The experiments continued for 55 consecutive days until they reached a stabilized condition. Hence, readings were taken after 55 days to evaluate the treatment efficiency, and the results of EBR and tubesettler were verified, as shown in Fig. 1. Also, the outcomes of this study will validate the enhancement capacity of the tube settler. American Public Health Association (APHA) standard testing methods were adopted for water sample analysis which was also adopted in similar studies^1,2^.

### Optimization of design parameters using response surface methodology approach

With the help of the Design-Expert software (version DX13.0.1), the experimental matrix is determined, where 20 experiments with different combinations of process variables are incorporated. Analysis of three process variables: pH, present time, and MLSS, were employed in the central composite design (CCD) model for understanding how chemical oxygen demand (COD), nitrate, and Phosphate removal percentages were affected. It has been observed that the sequential sum of squares test and lack of fit test were best suited and applied during the analysis in the response surface methodology approach (RSM) model. The software's optimization feature helps determine the best values in existing systems^28,29^. RSM approach is utilized to optimize operation parameters considering the three-factor and five-level CCD analysis. In the present study, input variables were optimized to maximize COD, nitrate, and phosphate, as shown in Table 3. The removal efficiencies for COD, nitrate, and phosphate are between 59.1 and 74.1%. Validation was done by calculating average experimental results based on optimum values provided by software optimization. There was a good match between theoretical and practical COD, nitrate, and phosphate removal values for RSM in improving the EBR process.

**Table 3:** Coded values are used in the CCD model.

Codes used	A: pH	B: Current time (min)	C: MLSS (mg L^−1^)
−1.0	6	20	2000
0.0	8	40	2500
1.0	12	60	3000

The First-order model, Grau second-order model, modified Stover–Kincannon model, and Monod model was used to investigate COD removal kinetics from the EBR reactor. For wastewater treatment employing biological systems, kinetic analysis methods are well-established. A steady state was reached following acclimatization, which necessitated the analysis. Models created from experimental data may be evaluated using ANOVA (Analysis of Variance). ANOVA provides statistical indicators such as the F-value and the P-value. F must be extensive for the model to be statistically significant, and the P-value must be below (0.05). High correlation coefficients are indicative of a reliable model.

## Results and discussion

**Figure 2:**
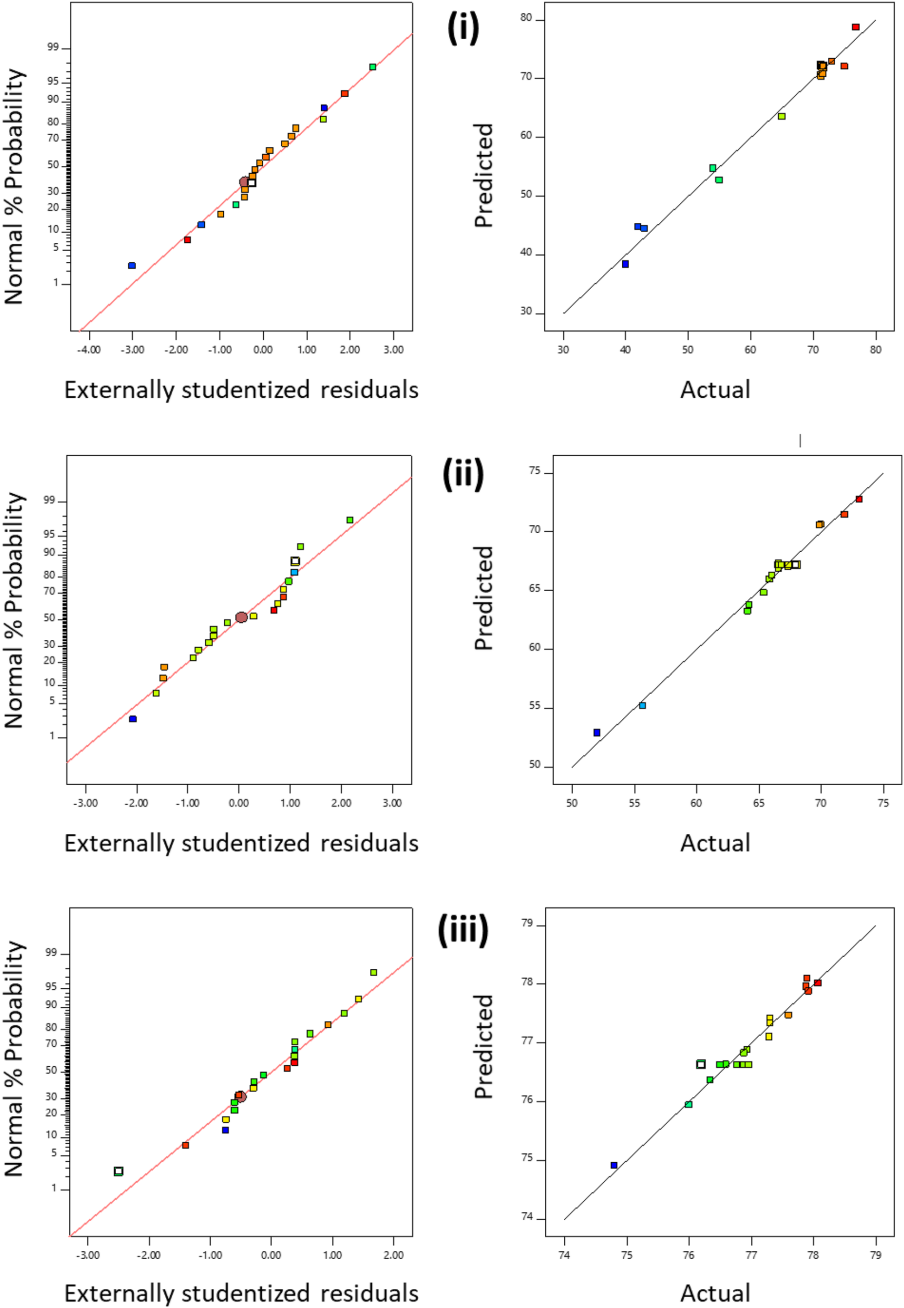
Normal probability versus studentized residuals and predicted versus actual plots for (**i**) COD removal, (**ii**) nitrate removal, and (**iii**) phosphate removal.

Considering the actual and predicted values, the model generated through the different inputted parameters should be diagnosed satisfactorily. It is pretty understanding that agreement between the actual and predicted values given the effectiveness and accuracy of the generated model, as shown in Fig. 2. The following polynomial regression model equations were obtained:

1 $$\begin{aligned} COD\;removal \, \% \, & = 76.63 - 0.019*A \, + \, 0.064*B \, - 0.511*C \, - 0.405*AB \, - 0.153*AC \, \\ &\quad - 0.099*BC \, + \, 0.263*A^{2} + \, 0.479*B^{2} - 0.303*C^{2} \\ \end{aligned}$$

2 $$\begin{aligned} Nitrate\;Removal \, \% \, & = 72.04 \, - 1.881*A - 0.142* \, B \, + \, 2.384*C \, + \, 2.623*AB \, + \, 8.579*AC \, \\ &\quad - 2.626*BC \, - 10.783*A^{2} + \, 0.223*B^{2} + \, 0.963*C^{2 } \hfill \\ \end{aligned}$$

3 $$\begin{aligned} & Phosphate \, Removal \, \% \, = \\ & 67.179 - 1.215*A \, + \, 3.539*B \, - 1.068*C \, + \, 1.610*AB \, - 2.559*AC \, + \, 0.392*BC \, + \, 0.788*A^{2} - 2.943*B^{2} + \, 0.564*C^{2} \\ \end{aligned}$$

where A is initial pH, B is current time (min), C is MLSS concentration (mg L^−1^) at which the study was carried out.

It has been observed that statistics for the model having low values represent well for the system and its predictions.

### Statistical analysis of COD, nitrate and phosphate removal

**Figure 3:**
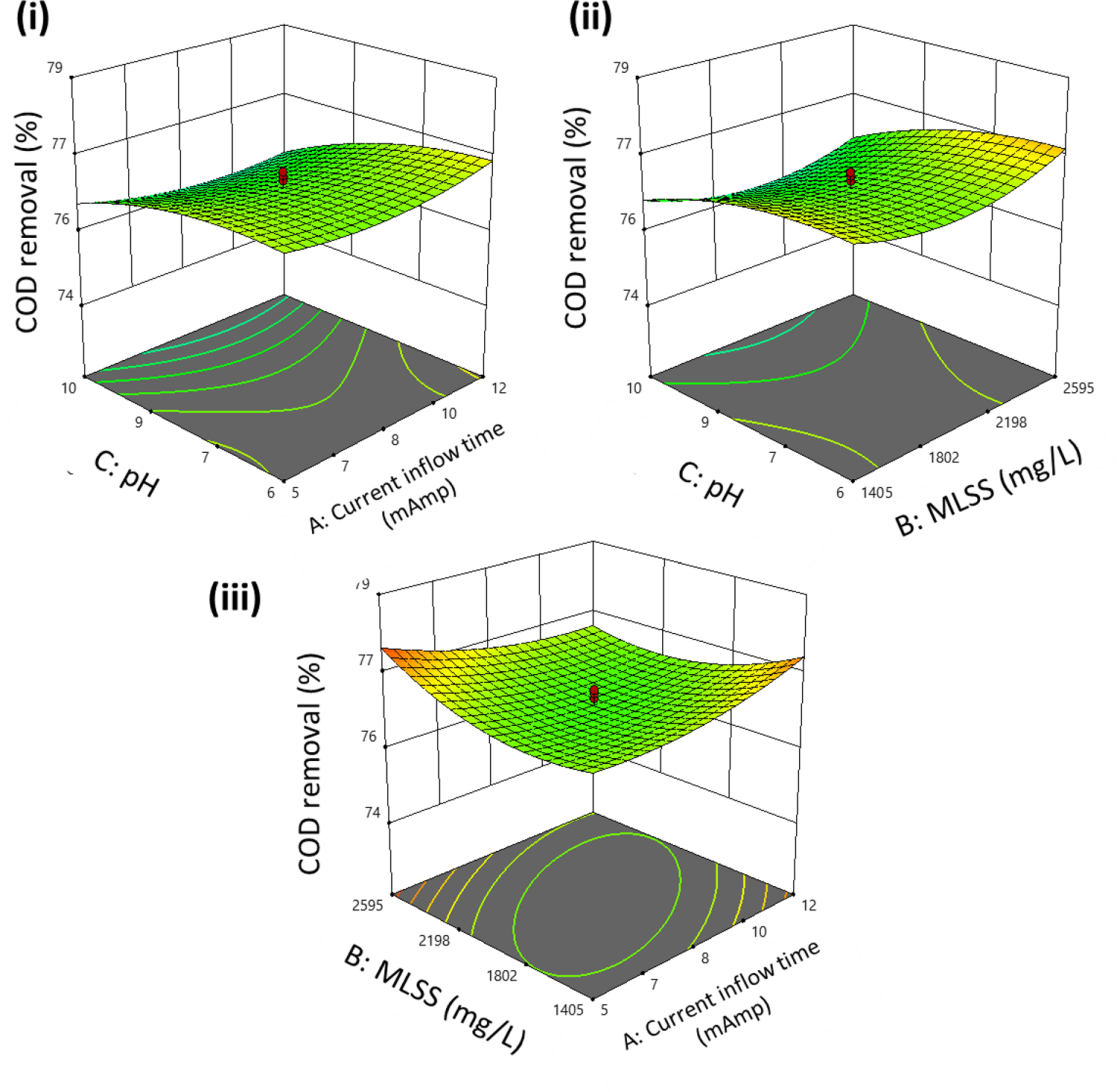
Model generated surface plot of % COD removal (**i**) pH versus current time (**ii**) pH vs. MLSS (**iii**) MLSS vs. current time.

**Figure 4:**
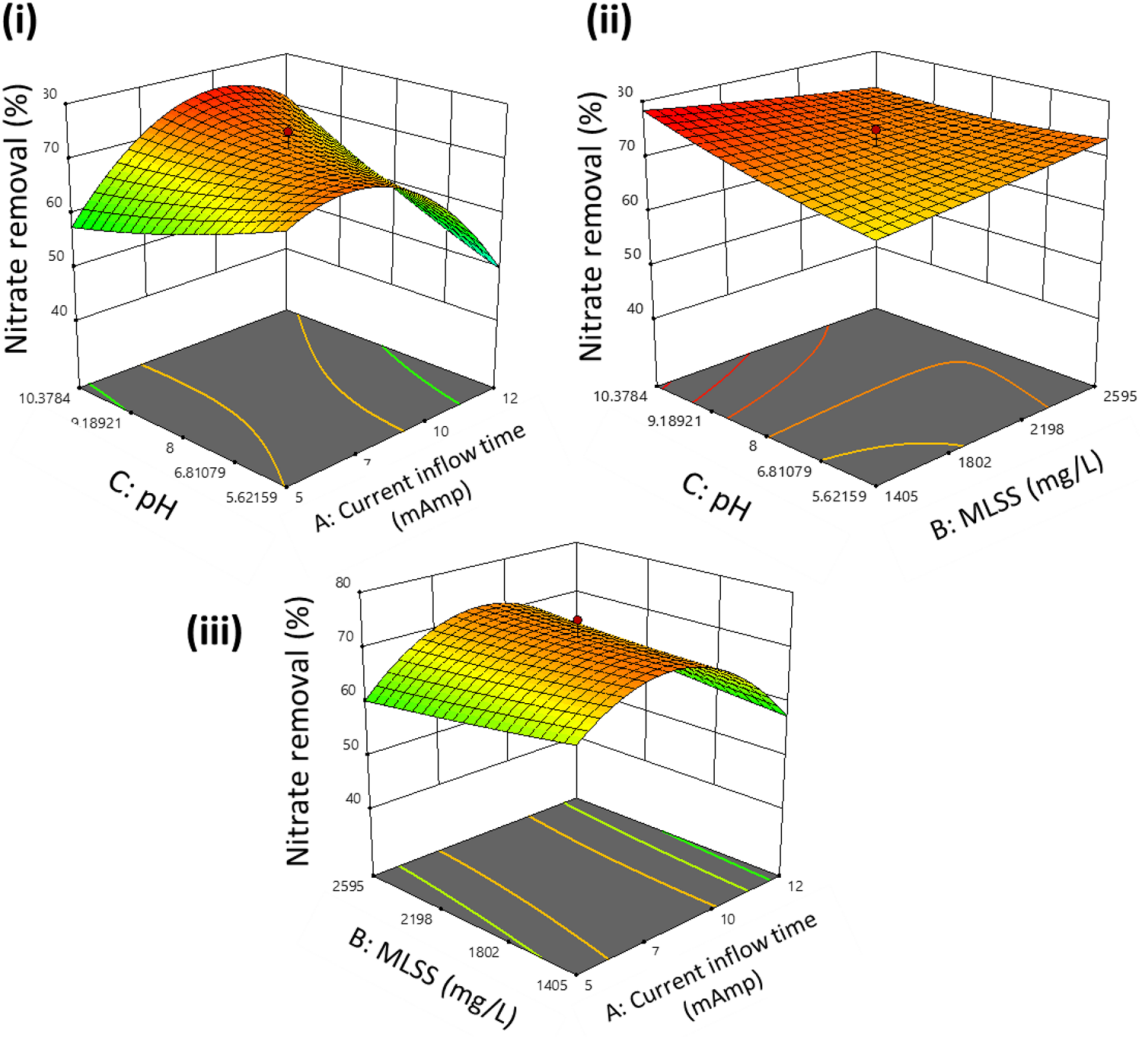
Model generated surface plot of %nitrate removal (**i**) pH versus current time (**ii**) pH vs. MLSS (**iii**) MLSS vs. current time.

**Figure 5:**
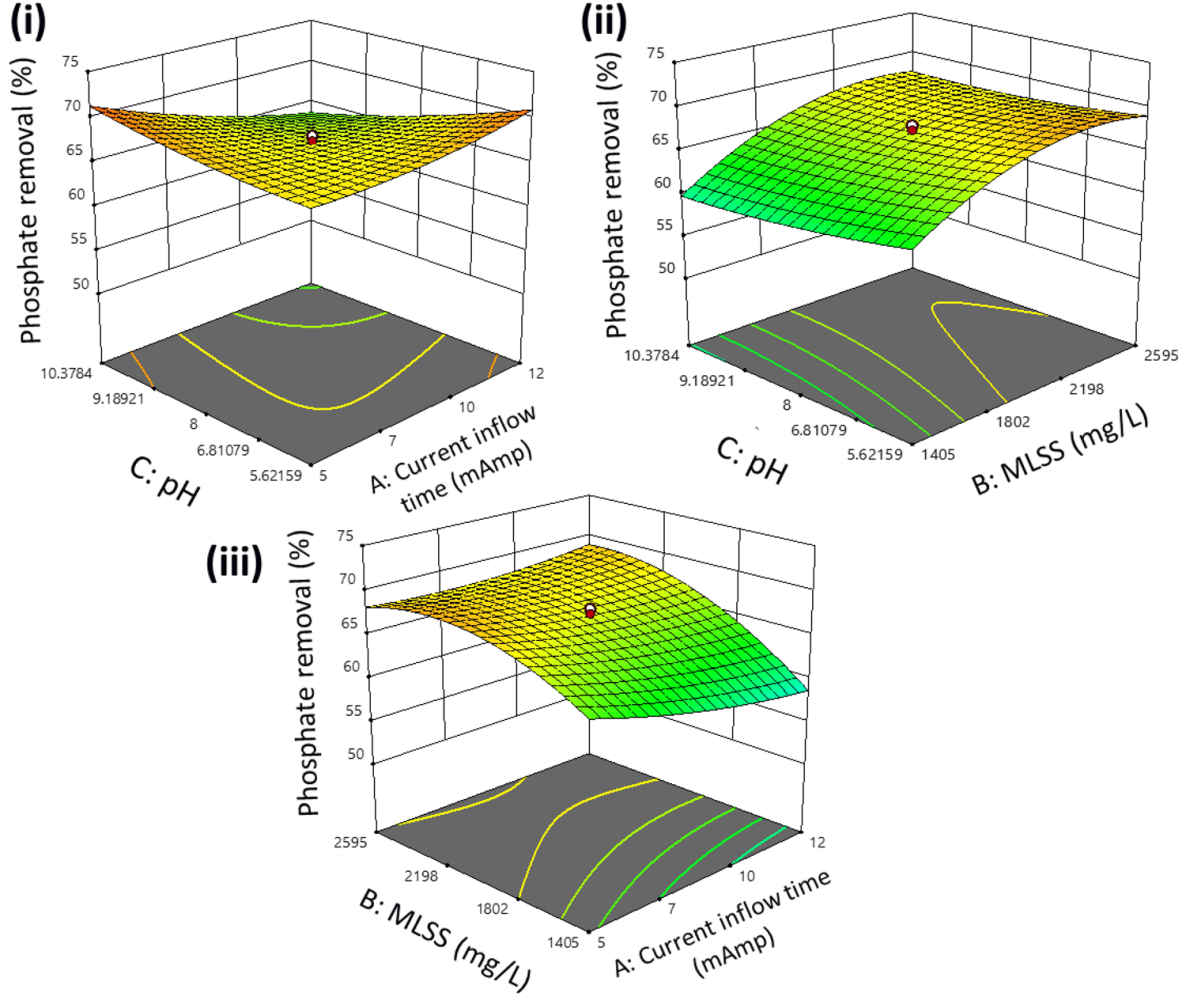
Model generated surface plot of %phosphate removal (**i**) pH versus current time (**ii**) pH versus MLSS (**iii**) MLSS versus current time.

It was seen that 3D surface plots could provide a better understanding of the interactive effects of the parameters. The 3D surface plots are illustrated in Figs. 3, 4, and 5, respectively. It was observed that the maximum removal efficiency for COD, nitrate, and phosphate is in the range of 59% to 74%.

Table 4 (i) shows the statistics for COD removal. Adeq Precision is desirable, which measures the signal-to-noise ratio and a ratio greater than 4. For the COD removal, Adeq Precision was 19.255, indicating an adequate signal. It was also observed that the adjusted R^2^ is 0.9118 (difference less than 0.2), and the predicted R^2^ of 0.8601 was significant, implying that the predictions are in good agreement with experimental values.

**Table 4:** Fit statistics for (i) COD removal, (ii) Nitrate removal, (iii) Phosphate removal.

**(i) COD removal**			
Std. Dev	0.2339	R^2^	0.9536
Mean	76.93	Adjusted R^2^	0.9118
C.V. (%)	0.3040	Predicted R^2^	0.8601
		Adeq Precision	19.2550
**(ii) Nitrate removal**			
Std. Dev	1.93	R^2^	0.9858
Mean	65.49	Adjusted R^2^	0.9730
C.V. (%)	2.95	Predicted R^2^	0.9164
		Adeq Precision	29.6081
**(iii) Phosphate removal**			
Std. Dev	0.8035	R^2^	0.9853
Mean	66.09	Adjusted R^2^	0.9720
C.V. (%)	1.22	Predicted R^2^	0.9165
		Adeq Precision	34.9452

Figure 3 illustrates the effect of current flow time and pH concerning the percentage removal of COD. The model predicted values observed were seen to lie in the range of 73.1% at MLSS values of 2500 mg L^−1^, keeping initial COD values as 200 mg L^−1^. As the COD load increases, it seems to be predicted that the overloading of bacteria occurs, thereby slowing down the consumption of organics. In Fig. 4, the expected removal efficacy shows upward trends with an increase in the values of MLSS, which also coincided with previous studies. As the value of MLSS increases, the contact time of biomass in the system increases, hence producing more effective results than others.

Table 4 (ii) shows the statistics for nitrate removal. The predicted R^2^ of 0.9164 was in reasonable agreement with the adjusted R^2^ of 0.9730. For the nitrate removal, Adeq Precision was 29.608, indicating an adequate signal. This model can be used to navigate the design space.

Table 4 (iii) shows the statistics for phosphate removal. The predicted R^2^ of 0.9165 was in reasonable agreement with the adjusted R^2^ of 0.9720. For the phosphate removal, Adeq Precision was 34.945, indicating an adequate signal. This model can be used to navigate the design space.

Figure 5 illustrates that as we reduce the cycle time from 24 to 18 h, the system efficacy, i.e., COD removal effectiveness shows a downward trend due to less contact time with biomass. Meanwhile, if we increase the cycle time, we observe higher efficacy in the system. The model generated surface plot in Fig. 5 illustrated that increasing MLSS values by 3000 mg L^−1^ will enhance the COD removal by 73.1%, keeping the initial pH constant. This may be due to many microbes that can break down organic matter. In aerobic reactors, pH is an essential factor in the growth of the microbial population. To create granules, the pH of the reactor has a direct impact. Studies have shown that granule formation occurs when bacteria grow at the ideal pH level, whereas mass proliferation of fungus occurs in an acidic environment.

### COD removal in EBR and tubesettler

**Figure 6:**
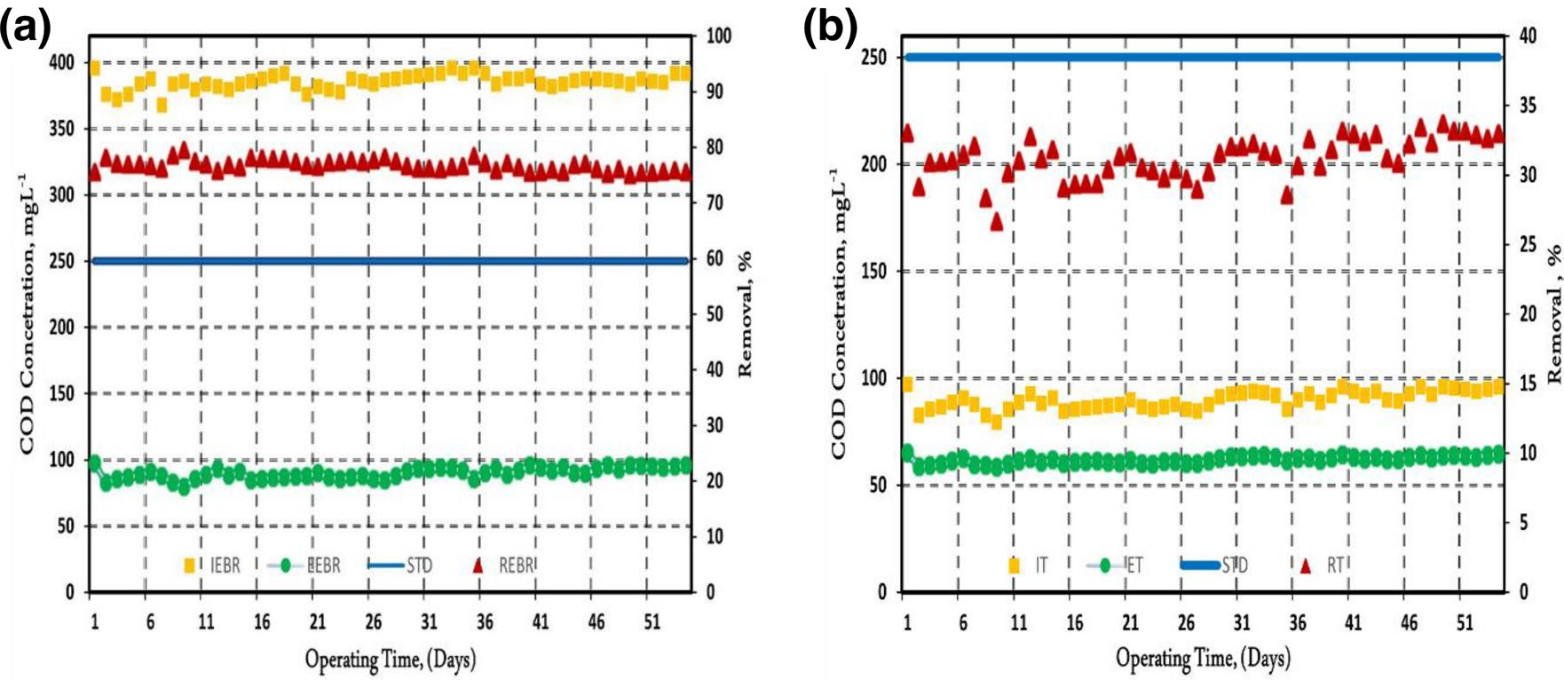
(**a**) Influent, effluent and removal of COD in EBR (IEBR = Influent Electrobioreactor, EEBR = Effluent Electrobioreactor, STD = Standard, REBR = Removal Electrobioreactor), (**b**) Influent, effluent, and removal of COD in tubesettler (IT = Influent tubesettler, ET = Effluent tubesettler, STD = Standard, RT = Removal tubesettler).

The Influence, effluent, and removal of COD in EBR & tubesettler are illustrated in Fig. 6a,b. Results demonstrate that the COD concentration is consistent and better COD removal efficacy rate. The average removal rate values observed in the EBR were between 74 and 79%, with the initial COD concentration kept around 360–396 mg L^−1^. It was also observed that tubesettler resulted in approximately 25–36% efficacy when the initial concentration was between 75 and 97 mg L^−1^. The results of EBR are promising and can be attributed to the fact that electrocoagulation takes place along with the oxidation and biodegradation process. It was also observed that the percentage removal of COD shows downward trends due to electrochemical oxidation and adsorption, thereby resulting in physical entrapment and electrostatic attraction^30^. It has also been reported in many other studies that COD removal of around 85–90% was observed using composite cathode membrane using MRB/MFC system^19^ for the specialized treatment of landfill leachate. It was seen with the electrooxidation process having COD removal of around 80–84% and 84–96% with submerged membrane bioreactors, using Iron electrode^6^. For the Coal industry, it was found to be around 85% using membrane electro bioreactors^31^.

In the current study, results seemed to be lower than the values reported in the previous studies. The main reason might be the employment of a modified EBR system and the production of biomass species. When the overall COD removal with tubesettler is considered, up to 83.58% removal efficiency is observed. The overall COD removal efficiency is significant and is at par with other studies^3–5^. This signifies that EBR performed better than tubesettler in COD removal. The tubesettler's lower removal efficiency can be attributed to lower influent concentration from already reduced wastewater from EBR.

### Nitrate removal in EBR and tubesettler

**Figure 7:**
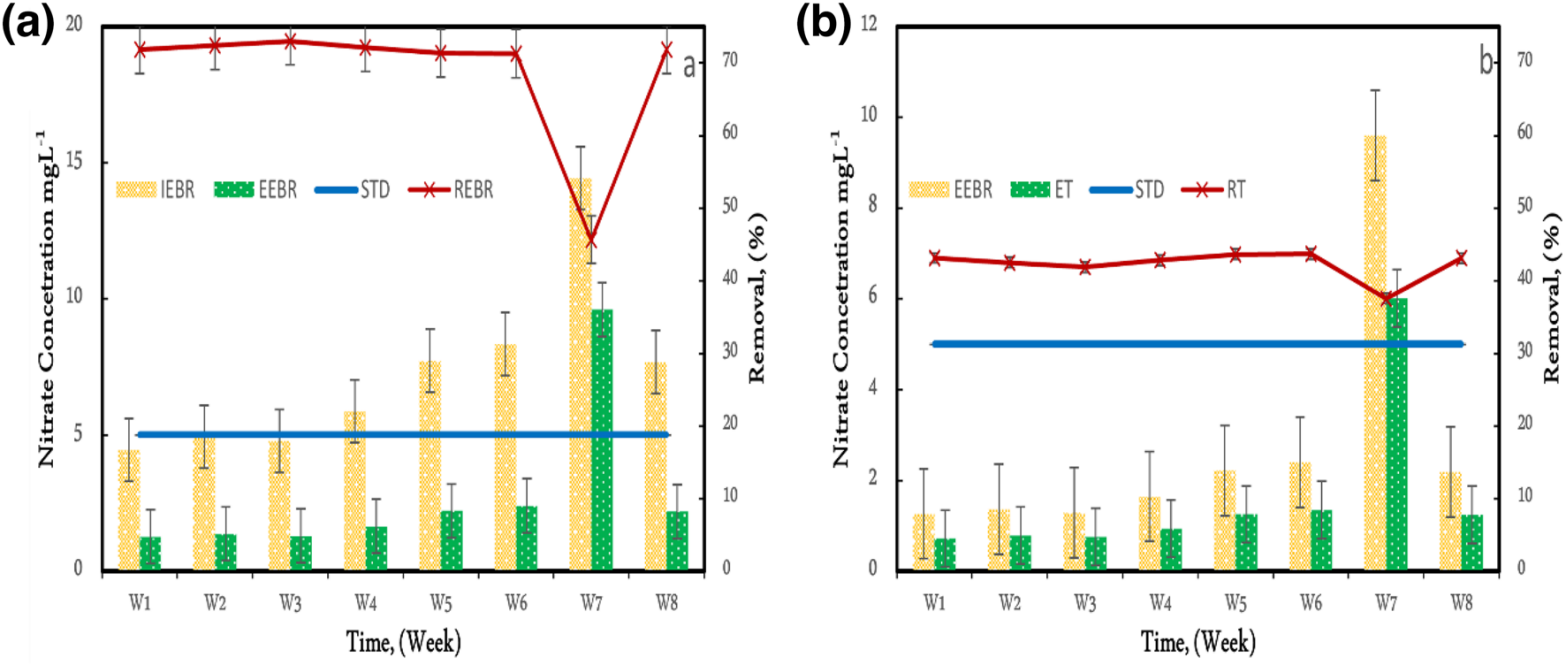
(**a**) Influent, effluent, and removal of nitrate in EBR (IEBR = Influent Electrobioreactor, EEBR = Effluent Electrobioreactor, STD = Standard, REBR = Removal Electrobioreactor), (**b**) Influent, effluent, and removal of nitrate in tubesettler (IT = Influent tubesettler, ET = Effluent tubesettler, STD = Standard, RT = Removal tubesettler).

It was observed in many studies that nitrifying is the leading cause of nitrification, i.e., conversion of NH_3_-N to nitrate NO_3_-N^10^. The indirect method of system nitrification process claudication was to be ascertained using measurements concerning ammonia values^32,33^. In the current study, the nitrification process was considered using the nitrate concentration measurement from the influent and effluent in both systems, i.e., EBR and tubesettler^34–36^. The nitrate concentration of influent and effluent was observed and illustrated in Fig. 7a,b. The system stabilized and produced enhanced results up to 70% of nitrate removal, and it was seen to be in the range of 40–45% for the tubesettler. It has been observed that EBR produced better results than the tubesettler. The results variation in both the systems were reasonably attributed mainly to two primary reasons (1) low influent concentration in the influent compared to the EBR system and (2) inhibition effect due to the applied DC field, which was absent in tubesettlers.

The removal efficiency of around 70% was achieved, lower than the values in submerged membrane bioreactors, i.e., 82%^6^. However, including a membrane would have enhanced the removal efficiency and considered a hybrid EBR system. The results of the current study are close enough to many other studies with a similar system and different operating parameters. Hence, a combined approach can be used for better efficacy. During the weekly analysis, the nitrate concentration during the 1st to 3rd week is lower than in the following weeks. As the concentration of nitrifying bacteria decreased, they had less to work with. Thus, the substrate concentration grew, and so did the removal rate. Nitrate concentrations rose by more than twice the previous week during Week 7. They slowed the bacterial activity, resulting in an efficiency decline to 47% from 70% during the last week's study period and weeks 6 and 8. A similar pattern emerged for the seventh week in a row in tubesettler. On the other hand, microorganisms overcame differences in engagement because the nitrate content was low in other weeks.

### Phosphate removal in EBR and tubesettler

**Figure 8:**
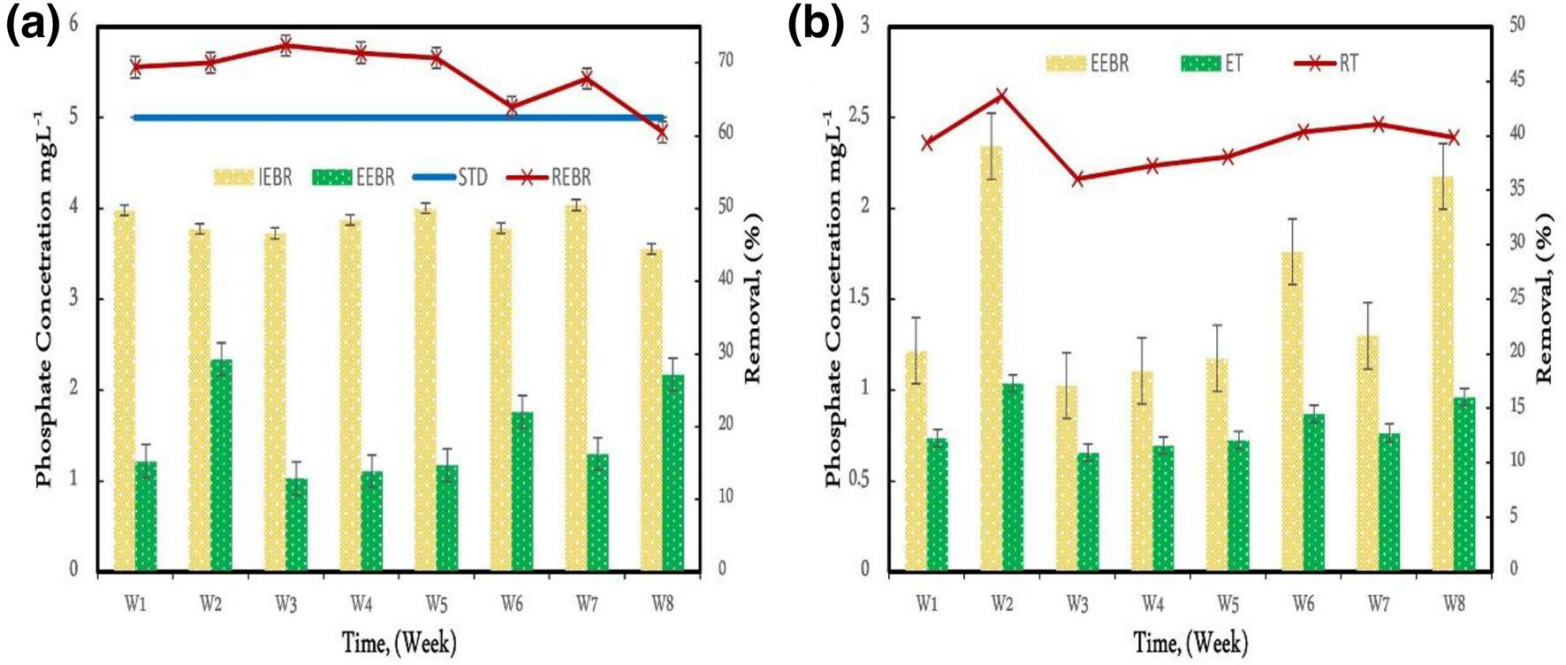
(**a**) Influent, effluent, and removal of phosphate in EBR (IEBR = Influent Electrobioreactor, EEBR = Effluent Electrobioreactor, STD = Standard, REBR = Removal Electrobioreactor), (**b**) Influent, effluent, and removal of phosphate in tubesettler (IT = Influent tubesettler, ET = Effluent tubesettler, STD = Standard, RT = Removal tubesettler).

Many researchers have looked at nitrate content, but none have looked at phosphate concentration. Eutrophication in receiving water bodies, on the other hand, is predominantly caused by phosphate and nitrate. Additionally, there is a lack of information available on hospital wastewater. The influent and effluent phosphate concentrations in the Electro bioreactor and the tubesettler is shown in Fig. 8a,b. A 75% reduction in the effluent phosphate content in EBR was achieved tubesettler had a 67% effectiveness in phosphate removal but a lower efficiency in nitrate reduction. A previous similar study that used a Submerged Membrane Electro bioreactor claimed a clearance rate of 76% to 95%, which is lower than this study's results^6^. Phosphate removal was reported at 50–70% using the electrocoagulation process for different Ph and current^6^.

In week 6 and week 8, the EBR's phosphate removal efficiency fluctuated dependent on the weekly average concentration in EBR. This volatility can be linked to a shift in the composition of hospital wastewater. tubesettler had a modest variation ranging from 5 to 6%. Although phosphate concentrations rose in week two, tubesettler removal efficiency improved. As demonstrated in Fig. 8a,b, the arriving wastewater ingredient exhibited a strong affinity in terms of phosphate reduction.

Excess effluent concentration and standard deviation from EBR and tubesettler are shown in Table 5. EBR performed better than tubesettler in COD reduction when nitrate and phosphate were compared. Because tubesettler solely employs a physical process to remove contaminants, this is to be anticipated. Effluent from the secondary treatment facility is sent to a tubesettler, which acts as a polishing unit. EBR eliminated COD by 91%, nitrate by 85%, and Phosphate reduction by 81% compared to tubesettler’ s total efficiency. At the same time, tubesettler reduced COD by 37%, nitrate by 51%, and phosphate by 53%. Hence, EBR primarily removed pollutants from wastewater while tubesettler acted as a polishing unit. Table 5 illustrates the effluent wastewater characteristics of EBR and tubesettler.

**Table 5:** Effluent wastewater characteristics of EBR and tubesettler.

Parameter	EBR	Tubesettler
COD (mg L^−1^)	89 ± 4.06	61 ± 1.6
Nitrate (mg L^−1^)	1.85 ± 0.54	1.05 ± 0.3
Phosphate (mg L^−1^)	1.34 ± 1.1	0.76 ± 0.31

### Kinetic models post optimization

#### First-order model

A first-order linear model was analyzed on the experimental data by plotting (So − Se)/Se against hydraulic retention time (HRT), providing K_1_ and R^2^. For COD, R^2^ values were 0.761 with a constant value of 1.213, as shown in Table 6. Henceforth based on the results, the obtained model did not seem to fit well for either of the cases.

**Table 6:** Analyzed kinetic models.

Parameter	Model analyzed	Equation used	Linearized equation	Kinetic parameters obtained		R^2^ value
COD	1st order	$\frac{{ - {\text{d}}s}}{{{\text{d}}t}} = \frac{Q}{v}\left( {s_{0} - s_{e} } \right) - k_{1} s_{e}$	$\frac{{s_{0} }}{{s_{e} }} - 1 = k_{1} HRT$	K_1_ = 1.213	–	0.761
	Grau 2nd order Model	$$\frac{{ - {\text{d}}s}}{{{\text{d}}t}} = \frac{{k_{2} xs_{e} 2}}{{s_{0}^{2} }}$$	$$\frac{{s_{0} }}{{s_{0} - s_{e} }}HRT = {\raise0.7ex\hbox{${HRT - S_{0} }$} \!\mathord{\left/ {\vphantom {{HRT - S_{0} } {k_{2} x}}}\right.\kern-\nulldelimiterspace} \!\lower0.7ex\hbox{${k_{2} x}$}}$$	–	K_s_ = 10^–05^	0.982
	Modified Stover-Kincanna model	$- \frac{{{\text{d}}s}}{{{\text{d}}t}} = \frac{Q}{v}\left( {\frac{{s_{0} - s_{e} }}{{s_{0} }}} \right) = \frac{{U_{M} \frac{{Qs_{0} }}{v}}}{{k_{B} + \frac{{Qs_{0} }}{v}}}$	$\frac{v}{{Q\left( {s_{0} - s_{e} } \right)}} = \frac{{k_{B} v}}{{U_{M} s_{0} Q}} + \frac{v}{{U_{M} }}$	K_B_ = 0.35	U_M_ = 1.73	0.978
	Monod Model	$- \frac{{{\text{d}}s}}{{{\text{d}}t}} = \frac{Q}{v}\left( {\frac{{s_{0} - s_{e} }}{{s_{0} }}} \right) = \frac{{U_{M} \frac{{Qs_{0} }}{v}}}{{k_{B} + \frac{{Qs_{0} }}{v}}}$	$\frac{vx}{{Q\left( {s_{0} - s_{e} } \right)}} = \frac{{k_{s} }}{{ks_{e} }} + \frac{1}{k}$	K = 0.062	K_s_ = 0.073	0.991

#### Grau second-order model

A Grau second-order model was analyzed on the experimental data by plotting HRT/((So − Se)/So) versus HRT. The COD constant obtained was K_s_ = 10^–5^, as shown in Table 6. The R^2^ value of 0.99 suggests a good correlation coefficient. Therefore, the obtained results fit well for AOX and COD.

#### Modified Stover–Kincannon model

Substrate utilization rate expressed as organic loading in this model is widely used in biological reactor kinetic modelling of wastewater. The developed model can evaluate the performance of the biological system and estimate its efficiency based on the input parameters. The kinetic constant K_B_ and U_max_ for COD were 0.35 and 1.73 g L^−1^ d^−1^, respectively. The R^2^ was 0.98 for the substrate removal, as presented in Table 6.

#### Monod model

COD utilization rate was obtained by plotting VX/Q (S_o_ − S_e_) against 1/S_e_. The value of 1/K (0.421) was obtained from the intercept, while the K_s_/K value (1.235) was the slope of the line. COD removal half-saturation values were 0.045 and 0.056 g L^−1^. These values infer a high affinity of bacteria for the substrate. The R^2^ value of 0.95 depicted an excellent correlation coefficient in the case of COD. The Monod model fits well for COD, resulting in R^2^ = 0.98, as shown in Table 6.

## Conclusions

This study investigated the performance of an EBR connected in series with a tubesettler to treat hospital wastewater. Based on the results obtained from 55 days of investigation, the Electro bioreactor efficiently improved the effluent quality of hospital wastewater. This study employed a novel combination system with tubesettler. The removal efficiency of EBR as an individual treatment system is low compared to other studies. Additionally, this combination has an advantage over different varieties as no additional filter, membrane, or chemicals are required, rendering it economical and more straightforward. Hence, tubesettler can successfully polish effluent quality from secondary treatment.

Further, this study also investigates phosphate and nitrate reduction from hospital wastewater. The optimized values for hydraulic retention time (HRT) for maximum COD removal was seen to be around 12 h with an MLSS concentration of approximately 2500 mg L^−1^. The results are interesting considering that it is counter-intuitive compared to the effects of HRT for higher removal of COD in the system. The decrease in the removal efficacy beyond 12 h was attributed to the F/M ratio reduction in the process, thereby resulting in the consumption of cell mass and hence lowering efficacy. Future studies are required to investigate high phosphate removal compared to nitrate reduction due to specific hospital constituents affecting nitrate removal. Also, more in-depth studies are needed to determine high phosphate removal despite a five times increase in influent concentration for hospital wastewater.

## Data Availability

The data supporting this study's findings are available from [Roohul Abad Khan]. Still, restrictions apply to the availability of these data, which were used under license for the current research, and so are not publicly available. However, data are available from the authors upon reasonable request and with permission of [Roohul Abad Khan].
